# Long-term water use efficiency and non-structural carbohydrates of dominant tree species in response to nitrogen and water additions in a warm temperate forest

**DOI:** 10.3389/fpls.2022.1025162

**Published:** 2022-11-07

**Authors:** Xiyan Jiang, Mengya Song, Yaqi Qiao, Mengzhou Liu, Lei Ma, Shenglei Fu

**Affiliations:** ^1^ Key Laboratory of Geospatial Technology for the Middle and Lower Yellow River Regions, College of Geography and Environmental Science, Henan University, Kaifeng, China; ^2^ Department of Forest Ecology and Management, Swedish University of Agricultural Sciences, Umeå, Sweden; ^3^ Henan Key Laboratory of Integrated Air Pollution Control and Ecological Security, College of Geography and Environmental Science, Henan University, Kaifeng, China

**Keywords:** nitrogen and water additions, water use efficiency, non-structural carbohydrates, nutrients stoichiometry, δ^13^C stable isotope

## Abstract

Nitrogen (N) deposition tends to accompany precipitation in temperate forests, and vegetation productivity is mostly controlled by water and N availability. Many studies showed that tree species response to precipitation or N deposition alone influences, while the N deposition and precipitation interactive effects on the traits of tree physiology, especially in non-structural carbohydrates (NSCs) and long-term water use efficiency (WUE), are still unclear. In this study, we measured carbon stable isotope (δ^13^C), total soluble sugar and starch content, total phenols, and other physiological traits (*e*.*g*., leaf C:N:P stoichiometry, lignin, and cellulose content) of two dominant tree species (*Quercus variabilis* Blume and *Liquidambar formosana* Hance) under canopy-simulated N deposition and precipitation addition to analyze the changes of long-term WUE and NSC contents and to explain the response strategies of dominant trees to abiotic environmental changes. This study showed that N deposition decreased the root NSC concentrations of *L. formosana* and the leaf lignin content of *Q. variabilis*. The increased precipitation showed a negative effect on specific leaf area (SLA) and a positive effect on leaf WUE of *Q. variabilis*, while it increased the leaf C and N content and decreased the leaf cellulose content of *L. formosana*. The nitrogen–water interaction reduced the leaf lignin and total phenol content of *Q. variabilis* and decreased the leaf total phenol content of *L. formosana*, but it increased the leaf C and N content of *L. formosana*. Moreover, the response of *L. formosana* to the nitrogen–water interaction was greater than that of *Q. variabilis*, highlighting the differences between the two dominant tree species. The results showed that N deposition and precipitation obviously affected the tree growth strategies by affecting the NSC contents and long-term WUE. Canopy-simulated N deposition and precipitation provide a new insight into the effect of the nitrogen–water interaction on tree growth traits in a temperate forest ecosystem, enabling a better prediction of the response of dominant tree species to global change.

## Introduction

Atmospheric nitrogen (N) deposition, an important threat to plant biodiversity, is mainly derived from natural processes and anthropogenic activities (Sala et al., 2000; Payne et al., 2017). Galloway (2004) predicted that, by 2050, global N deposition would double that of the early 1990s (Galloway et al., 2004). A modest rate of N deposition boosts forest abundance and foliar growth (Mao et al., 2018). However, excessive N deposition can lessen the diversity of plant species (Stevens, 2004; Schrijver et al., 2011; Payne et al., 2017), threaten plant growth (Galloway et al., 2008; Mao et al., 2018), weaken plant resistance (Bobbink et al., 2010), and even change the community structure of forests (Song et al., 2017). Additionally, temperate forests are regarded as N-limited, and N deposition affects the carbon (C): N: phosphorus (P) balance of forest vegetation and the C cycle process (Thomas et al., 2010). Generally, N deposition and precipitation occur simultaneously under natural conditions (Jia et al., 2014). Thus, the research of N-precipitation interaction is more accord with natural status. Precipitation affects the changes in belowground and aboveground communities, and its intensity is a critical factor in habitat alteration—for example, increasing water can increase vegetation production (Fu et al., 2018) and decrease the conductance of leaves (Sellin et al., 2019).

The long-term water use efficiency (WUE), which can be regarded as a comprehensive index of plant growth suitability under water stress, is generally expressed by the ratio of photosynthesis and stomatal conductance (Eamus, 1991; Jennings et al., 2016). Previous studies have shown that WUE is related to average precipitation, the length of plant growing seasons, and the specific leaf area (SLA) (Wright et al., 1994; Xiao et al., 2013; Easlon et al., 2014). N application can promote plant WUE (Martin et al., 2010; Zhang et al., 2018; Hu et al., 2019). The impact of rainfall on WUE is complex, and there are differences in WUE among various plant species (Xiao et al., 2013). Some studies have shown that WUE increases with water deficit (Ying et al., 2015) and decreases with increasing precipitation (Niu et al., 2011; Huang et al., 2017). Moreover, the WUE has been used to describe the coupling relationship between vegetation productivity and carbon supply and water consumption, which is an important part of the coupling cycles of carbon–nitrogen–water (Felzer et al., 2011; Yu et al., 2014).

Non-structural carbohydrates (NSCs) are crucial substrates for plants’ growth and metabolism, which consist of total soluble sugar and starch, and both are from photosynthesis (Hartmann and Trumbore, 2016; Hao et al., 2021). They participate in many important metabolic processes (*e*.*g*., photosynthesis and respiration), while non-metabolic processes include osmotic regulation, vascular transport, and cold tolerance (Sala et al., 2012; Dietze et al., 2014). Plant carbon dynamics can be inferred *via* quantifying the changes in NSC concentrations (Mitchell et al., 2013). When plant carbon demand exceeds supply, the starch in plant organs decomposes into soluble sugars, providing energy for and maintaining the growth of plants (Dietze et al., 2014; Hartmann and Trumbore, 2016). When carbon supply is over demand, more NSCs are stored for later use (Hoch et al., 2003; Würth et al., 2005; Smith and Stitt, 2007). Soluble sugars are indispensable for the metabolism, osmotic regulation, and energy conversion of plants. The soluble sugar content would increase for osmotic regulation to relieve stress under external stress (Dietze et al., 2014; Hao et al., 2021). Starch is an effective and relatively stable storage molecule that not only provides energy and sugar but also promotes plant growth (Dietze et al., 2014; Schiestl-Aalto et al., 2019). The NSC concentration reflects the adaptability of plants to a changeable environment (Hoch et al., 2003; Mo et al., 2020). When the NSC content is depleted, plants would face death (Hartmann and Trumbore, 2016; Santos et al., 2021). When the external environment changes, such as N deposition and increasing precipitation, the content of NSCs would change (Li et al., 2018; Du et al., 2020). N application reduces the soluble sugar concentration in leaves and roots (Wang et al., 2019) and decreases the starch and NSC concentrations in roots under high N deposition (Li et al., 2018).

WUE reflects the carbon–water relationship (*i*.*e*., photosynthesis and respiration), and NSCs reflect the balance between carbon demand and carbon supply (Smith and Stitt, 2007). The carbon stable isotope (δ^13^C) is the most common method for determining the long-term WUE (Farquhar and Richards, 1984; Farquhar et al., 1989; Livingston et al., 1999; Saurer et al., 2004; Easlon et al., 2014; Wang et al., 2018). The amount of C, N, and P in leaves represents the amount of nutrients that were accessible to plants and had a significant effect on plant growth (Li et al., 2008). The stoichiometric ratios of C, N, and P reflect the interaction between plants and the environment (Gruber and Galloway, 2008), which are important ways to know the reason of change of NSCs and WUE. In addition, N and P contents were the main limiting factors for plant growth, which not only affected species richness but also influenced biochemical cycles. Moreover, leaf NSC variables and C:N:P stoichiometric variables exhibit a substantial association (Xie et al., 2018). NSCs are the product of photosynthesis, and WUE is the ratio of photosynthetic rate to stomatal conductance, both of which reflect plant photosynthetic capacity. Although there have been many studies on the response of NSCs and WUE to external environmental disturbances, relatively few studies have been conducted on how NSCs and WUE respond to canopy N–water interaction.

The traditional method is mainly to add N directly to the understory, but this ignores the N interception effect of the forest canopy (Tian et al., 2019; Tang et al., 2020). In this experiment, we selected two dominant tree species in a temperate forest to conduct multi-year canopy N addition and precipitation enhancement, and we determined the effect of N and water addition on NSCs and leaf long-term WUE in the dominant tree species in the growing season. We hypothesized that (1) increasing the precipitation and N addition alone would decrease the leaf WUE (1-year period) of *Q. variabilis* and *L. formosana*, and the N–water interaction would have no significant effect on leaf WUE; (2) N addition has different effects on leaf NSCs and root NSCs of the same tree species; and (3) increasing the precipitation could alleviate the effects of N deposition on leaf WUE and NSCs.

## Materials and methods

### Study site

This study was conducted at the Henan Dabieshan National Field Observation and Research Station of Forest Ecosystem (31°51′ N, 114°05′ E), which is a natural, deciduous, and broad-leaved mixed forest in Henan Province, China. The field station was located in the transitional zone between the subtropical and warm–temperate climates. The mean annual temperature is 15.2°C, and the mean annual precipitation is 1,119 mm; the background rate of N deposition in atmospheric precipitation is approximately 19.6 kg N ha^-1^ year^-1^ in this region (monitoring data from August 2010 to July 2011). *Quercus variabilis* Blume and *Liquidambar formosana* Hance are the dominant tree species of the forest ecosystem. The forest is about 45 years old and has yellow–brown loam soil (Zhang et al., 2015).

### Experimental design

The setting of the experimental platform comprehensively considered all factors (*e*.*g*., vegetation and slope). We chose two dominant tree species in this forest: *Q. variabilis* and *L. formosana*. Both of them are dominant tree species in the temperate forest ecosystem and grow up to 30 m tall. *Q. variabilis* is shade tolerant with well-developed root systems, and *L. formosana* is modestly shade tolerant and drought resistant. This experiment was designed using random distribution within four blocks, and each block was set randomly with four different treatments, namely: (1) control treatment (CK, ambient environment and without N addition), (2) canopy N addition with 50 kg N ha^-1^ year^-1^ (CN), (3) canopy water addition with increasing 30% greater than local mean annual precipitation (CW), and (4) interaction of canopy N application (50 kg N ha^-1^ year^-1^) and water addition (30% of local annual precipitation) (CWN). The control (CK) plots were squares with a side length of 30 m, and the total area of each plot was about 900 m^2^. The canopy treatments (CN, CW, and CWN) were circular plots with a diameter of 34 m, and the total area of each plot was about 907 m^2^. A triangular iron tower with a height of 35 m was built in the center of each circular quadrat, about 5 m above the forest canopy, and a set of rocker nozzles was installed on top of the tower to drive the rocker nozzles to rotate 360° to ensure the uniformity and accuracy of spraying and accessibility (Zhang et al., 2015; Zhao et al., 2020; **Supplementary Figure S1**). The experiment began in April 2013, and the CN treatment was administered monthly from May to mid-October and repeated annually. The CW treatment was performed weekly at the same time. In August 2018, we randomly selected six healthy and sunny leaves of each dominant tree species from all treatments. Fine root samples (diameter <2 mm) of the two dominant tree species (*Q. variabilis* and *L. formosana*) in all plots were collected from branch roots and washed with purified water. The leaf and root samples were placed in labeled paper bags, dried in the oven at 70°C for 48 h, and analyzed in the laboratory. The basic soil properties of these four locations are shown in **Supplementary Table S1**.

### Long-term water use efficiency analysis

δ^13^C was determined in the sample leaves collected from each treatment, and the samples were placed in an oven (70°C for 48 h) and ground into powder with a ball mill. The δ^13^C analysis was performed using 5 mg of sample leaves, which were burned in an elemental analyzer (Vario EL, Elementar, Hanau, Germany) and analyzed using an isotope ratio mass spectrometer (Finnigan Mat, typr Deltas). The relationship between carbon isotopic composition and WUE was determined using the equations in the following discussion.

Following Farquhar et al. (1989), the discrimination of isotopic composition was defined as:


(1) $$\Delta =\left(\delta^{13}{\text{C}}_{\text{a}}-\delta^{13}{\text{C}}_{\text{p}}\right)/(1+\delta^{13}{\text{C}}_{\text{p}}/1,000)$$

where δ^13^C_a_ represents the isotopic composition of atmospheric CO_2_, δ^13^C_p_ is the CO_2_ isotope value of plant leaves, and discrimination expresses the discrepancy between the δ^13^C values of atmospheric CO_2_ and plants to some extent. Farquhar et al. (1989) illustrated the relationship between △ and C_p_/C_a_ as follows:


(2) $$\Delta =\text{a}+\left(\text{b}-\text{a}\right)\left({\text{C}}_{\text{p}}/{\text{C}}_{\text{a}}\right)$$

where C_p_/C_a_ is the ratio of intercellular to environment CO_2_ concentrations, a (=4.4‰) represents fractionation due to air diffusion, and b (=27‰) is net fractionation due to carboxylation. Equation (2) can be converted into equation (3) as follows:


(3) $${\text{C}}_{\text{p}}={\text{C}}_{\text{a}}(\Delta -\text{ a})/\left(\text{b}-\text{a}\right)$$

W: WUE, the ratio of net photosynthesis A to stomatal conductance for water vapor g_s_, was used following Saurer et al. (2004):


(4) $$\text{W}=A/g_{s}=\left({\text{C}}_{\text{a}}-{\text{C}}_{\text{p}}\right)/1.6$$

where 1.6 is the ratio of the CO_2_ and water vapor diffusivity in the air. Equations (2) and (4) were combined to obtain the relationship between WUE, W, and isotopic discrimination, △:


(5) $$\text{W}={\text{C}}_{\text{a}}(\text{b}-\Delta )/1.6\;\left(\text{b}-\text{a}\right)$$

The relevant items were determined according to this formula, and WUE was calculated using the carbon isotopic composition.

### Determination of non-structural carbohydrate content in leaves and roots

NSC content was determined using the colorimetrical phenol−sulphuric acid method (Yemm and Willis, 1954; Dubois et al., 1956). In the study, the sum of total soluble sugar and starch was regarded as the total amount of NSCs. Briefly, the total soluble sugar content was determined by weighing 50 mg of dry fine power into centrifuge tubes, which was added with 5 ml of 80% ethanol, incubated in a water bath at 80°C for 30 min, and then centrifuged at 7,000 *g* for 5 min. The supernatants were transferred to centrifuge tubes. This procedure was repeated twice, and the supernatants were pooled together; then, colorimetric analysis was performed for measuring the soluble sugar. The residue was used for measuring the starch content. The solid residues left in tubes after total soluble sugar extraction were oven-dried at 80°C (24 h). Then, 2 ml water was added, and the samples were boiled in boiling water for 15 min. Subsequently, 2 ml of 9.2 M perchloric acid was added, and supernatants were collected after centrifuging at 7,000 *g* for 10 min. Then, the solution was used to measure the starch content.

### Determination of C, N, and P content in leaves

A mesh with a pore size of 250 µm was used to crush the dried biomass of samples into fine powder. The dry powdered sample (0.5 g) was used to determine the C, N, and P content using fast dichromate oxidation, an elemental analyzer (Vario EL, Elementar, Hanau, Germany), and induced plasma emission spectroscopy (Hötscher and Hay, 1997), respectively. To calculate the C:N:P stoichiometry relationship, the elemental leaf C/N ratio and N/P ratio were calculated.

### Determination of total phenols, lignin, and cellulose content in leaves

The total phenol content in the leaves was determined using the Folin–Ciocalteu reagent method as described by Khokhar and Magnusdottir, 2002 and Gundale et al. (2010), as the Folin-Ciocalteu reagent reacts with total phenol to produce a blue color that represents the total phenol content and can be determined spectrophotometrically. The leaf lignin and cellulose contents were measured according to the National Renewable Energy Experimental Procedures using two-step acid hydrolysis methods (Sluiter et al., 2008; Hou et al., 2020). A Muffle furnace (Neytech3-550; Lab-Pro Inc., Sunnyvale, CA, USA) and UV–vis spectroscopy TU-1901 (Purkinje General Instruments Ltd., Beijing, China) were used after the separation of leaf lignin into soluble and insoluble acids. Leaf cellulose was quantified using high-performance liquid chromatography Agilent-1260 (Agilent Technologies, Santa Clara, CA, USA).

### Determination of specific leaf area

The SLA was determined by dividing the single-sided area of a single leaf by its dry weight using the leaf area analysis system to read the single-sided area of each leaf. The dry weight of a single leaf was measured after being placed in a drying box at 70°C for 48 h (Ramalho et al., 2013).

### Determination of chlorophyll a and b content

The chlorophyll content was measured using spectrophotometry (Wang et al., 2015), following the protocol of Lichtenthaler (1987). Each sample (0.5 g) was extracted and ground into slurry, and 80% (v/v) acetone solution was used to extract the chlorophyll. Absorbance was measured using a spectrometer (Unicam UV-330, Unicam, Cambridge, UK) at a certain wavelength (Chl a at 663 nm and Chl b at 646 nm).

### Data analyses

We used control variables and comparison methods to conduct experimental processing and conducted the model analysis to verify the hypotheses. The three-way analyses of variance (ANOVA) were used to evaluate the effects of species, N addition, and water addition on physiological properties. One-way ANOVA with the least significant difference (*P<* 0.05) was used to analyze the differences among CK, N addition, water addition, and N–water interaction treatments on the concentration of NSCs in leaves and roots, C:N:P stoichiometry, δ^13^C, and other indices. A correlation analysis was used to analyze the relationship between WUE and NSCs of *Q. variabilis* and *L. formosana*. Principal component analysis (PCA) of the two dominant trees’ physiological traits was used to show the most discriminatory changes under canopy N and water addition. Data analysis was performed in SPSS 26.0 (SPSS Inc., Chicago, IL, USA) and Canoco 5.0 (Microcomputer Power, Ithaca, NY, USA).

## Results

### Changes in leaf C:N:P stoichiometry under N and water additions

C:N:P stoichiometry in leaves was affected by the treatments and species (**Table 1**). Leaf C, N, and P content and their stoichiometry showed no significant change across the treatments in *Q. variabilis*. For *L. formosana*, canopy N addition (CN) increased the leaf C/N ratio. The leaf C and N content of *L. formosana* was significantly increased, but the leaf C/N ratio was decreased under canopy water addition (CW) and water–nitrogen interaction (CWN) treatments. There was no difference in leaf P content and C/P ratio between the two species under CK treatment however, which showed an opposite trend between *Q. variabilis* and *L. formosana* under the treatments. The responses of leaf C, N, and P to N addition and rainfall enhancement were different, and the differences in leaf C:N:P stoichiometry of the two tree species showed different strategies of resource utilization.

**Table 1:** Changes in leaf C, N, and P content and C:N:P stoichiometry of *Q. variabilis* and *L. formosana* under canopy N application and water addition conditions.

Species	Treatments	LTC (mg g^-1^)	LTN (mg g^-1^)	LTP (mg g^-1^)	Leaf C/N ratio	Leaf C/P ratio	Leaf N/P ratio
*Q. variabilis*	CN	508.18 ± 9.70 abc	18.03 ± 0.92 a	1.44 ± 0.24 ab	28.25 ± 1.10 c	361.91 ± 58.94 ab	12.87 ± 2.34 bc
	CW	501.86 ± 13.52 abc	17.75 ± 1.01 a	1.17 ± 0.08 b	28.33 ± 0.88 c	431.98 ± 32.92 ab	15.27 ± 1.35 ab
	CNW	512.30 ± 5.35 ab	18.6 ± 1.15 a	1.11 ± 0.09 b	27.65 ± 1.56 c	466.00 ± 40.35 a	16.92 ± 1.79 a
	CK	497.39 ± 8.77 abc	17.65 ± 2.08 a	1.22 ± 0.12 ab	28.51 ± 2.75 c	411.88 ± 44.04 ab	14.56 ± 1.79 ab
*L. formosana*	CN	489.60 ± 10.76 c	13.66 ± 0.51 b	1.08 ± 0.06 b	35.92 ± 1.92 a	455.35 ± 25.56 a	12.72 ± 1.11 bc
	CW	515.72 ± 13.64 a	18.01 ± 0.92 a	1.44 ± 0.15 ab	28.68 ± 0.73 c	360.95 ± 37.64 ab	12.58 ± 1.20 bc
	CNW	513.87 ± 8.32 a	17.70 ± 0.58 a	1.65 ± 0.17 a	29.05 ± 0.71 c	314.38 ± 29.22 b	10.83 ± 1.03 c
	CK	493.56 ± 11.40 bc	15.27 ± 1.31 b	1.36 ± 0.20 ab	32.45 ± 1.96 b	371.05 ± 56.30 ab	11.46 ± 1.79 c
	F_S_	ns	***	*	***	*	***
	F_N_	ns	ns	ns	ns	ns	ns
	F_W_	**	***	ns	***	ns	ns
	F_S × N_	ns	ns	ns	*	ns	ns
	F_S × W_	*	**	***	***	**	*
	F_N × W_	ns	ns	ns	ns	ns	ns
	F_S × N × W_	ns	ns	**	ns	**	*

Values followed by the same letter in the same column are not significantly different at P< 0.05 according to least significant difference test. The values are expressed as means ± SE (n = 4). The significance values of the factorial analysis (ANOVA) are shown as follows: ns, not significant; *0.01< P ≤ 0.05; **0.001< P ≤ 0.01; ***P ≤ 0.001.

CK, control group; CN, canopy N addition; CW, canopy water addition; CNW, canopy N and water addition; LTC, leaf total carbon; LTN, leaf total nitrogen, LTP, leaf total phosphorus; F_S_, species effect; F_N_, nitrogen effect; F_W_, water effect; F_S × N_, species × nitrogen effect; F_S × W_, species × water effect; F_N × W_, nitrogen × water effect; F_S × N × W_, species × nitrogen × water effect.

### Changes in long-term WUE under N and water additions

**Figure 1:**
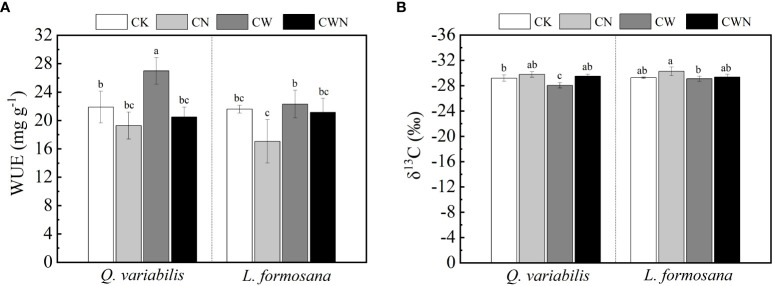
Response of long-term water use efficiency **(A)** and stable isotope carbon **(B)** of two tree species under canopy N and water additions. Values are means ± SE (n=4). Different letters indicate that the differences in means among the treatments are significant (*P <* 0.05).

The response trends of the WUE of the tree species to N and water addition were consistent (**Figure 1**). CN significantly decreased the WUE and δ^13^C of *L. formosana* and reduced the WUE of *Q. variabilis.* The WUE and δ^13^C of *Q. variabilis* significantly increased in CW (**Figures 1A, B**). Compared with *L. formosana*, the WUE of *Q. variabilis* was more impressionable under CW condition. The WUE of the two trees showed no obvious differences under CWN (**Figure 1A**).

### Changes in non-structural carbohydrates under N and water additions

**Figure 2:**
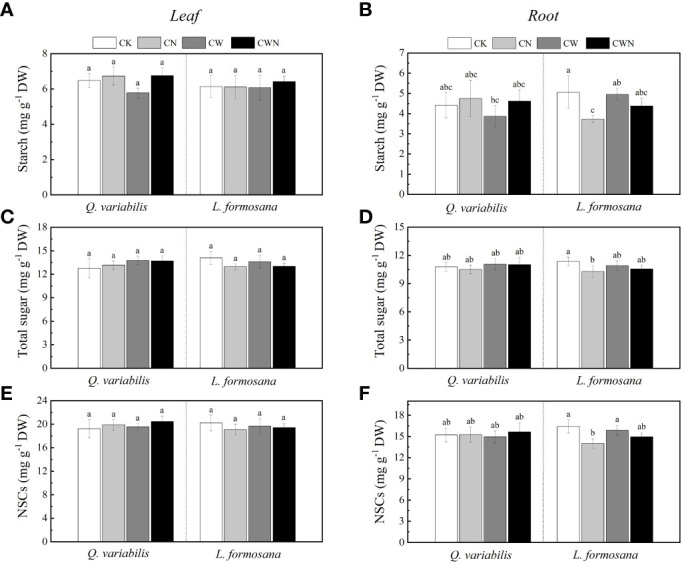
Changes in leaf and root starch **(A, B)**, leaf and root total soluble sugar **(C, D)**, leaf and root NSCs **(E, F)** concentration of two tree species under canopy N and water additions. Values are means ± SE (n=4). Different letters indicate that the differences in means among the treatments are significant (*P <* 0.05).

The leaf NSCs of tree species had no change among the four treatments but showed differences between leaf and root responses to all treatments. CN had a significantly negative effect on the root NSCs, starch, and total sugar of *L. formosana* (**Figures 2B, D, F**). The leaf NSCs, starch, total sugar, and root NSCs of the two tree species were not significantly affected by CW and CWN (**Figures 2 A, C, E, F**). Compared with CK, the leaf and root starch content decreased in CW for *Q. variabilis*. The root starch and total sugar contents of *L. formosana* were lower in CN, CW, and CWN treatments than in CK (**Figure 2**). N application and increased rainfall affected the total sugar and starch in the leaves and roots, but the total NSCs remained relatively stable.

### Changes in leaf chlorophyll a and b under N and water additions

**Figure 3:**
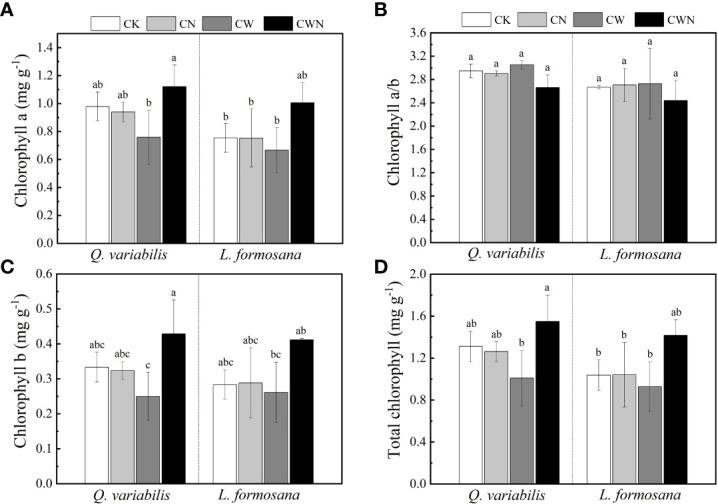
Effects of leaf chlorophyll a **(A)**, chlorophyll a/b **(B)**, chlorophyll b **(C)**, and total chlorophyll **(D)** of two tree species under canopy N and water additions. Values are means ± SE (n=4). Different letters indicate that the differences in means among the treatments are significant (*P <* 0.05).

The N and water interaction obviously affected the leaf chlorophyll a and b of *Q. variabilis* and *L. formosana* (**Figures 3A–D**). CN did not affect the leaf total chlorophyll (Chl a + b) content of the two trees species, but CW decreased the leaf total chlorophyll content of *Q. variabilis* (**Figure 3D**). In contrast to CK, the leaf total chlorophyll content of the two tree species was higher under CWN treatment. CWN had the greatest leaf total chlorophyll content than that in CK, CN, and CW treatments, and the total chlorophyll content of *Q. variabilis* was higher than that of *L. formosana* (**Figure 3D**). The leaf chlorophyll b content of the trees was lower than that of chlorophyll a in all treatments (**Figures 3A, B**).

### Changes in specific leaf area and total phenol, lignin, and cellulose content

**Figure 4:**
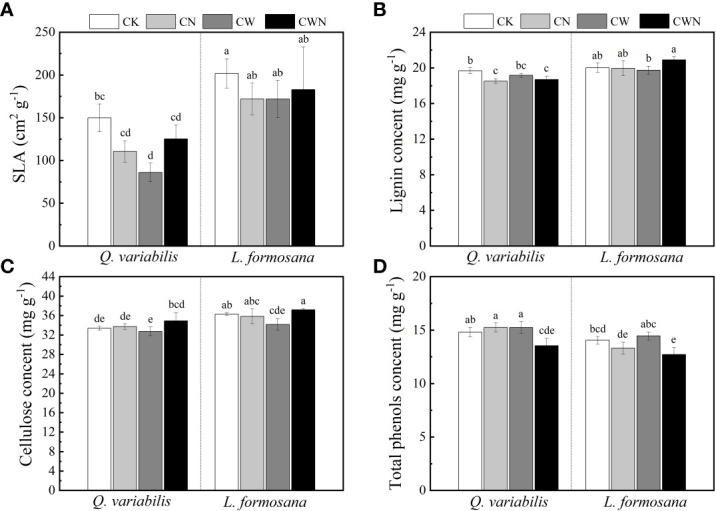
Effects of specific leaf area **(A)**, lignin **(B)**, cellulose **(C)**, and total phenol content **(D)** of two tree species under canopy N and water additions. Values are means ± SE (*n* = 4). Different letters indicate that the differences in means among the treatments are significant (*P<* 0.05).

The SLA of the two tree species was lower under CW than that of CK treatments, and CW significantly decreased the SLA of *Q. variabilis* (**Figure 4A**). CN and CWN significantly reduced the leaf lignin content of *Q. variabilis*. The leaf lignin content of *L. formosana* differed between CW and CWN (**Figure 4B**). CW obviously decreased the leaf cellulose of *L. formosana*, and the leaf cellulose content of the two trees was lower in CW treatment than in CWN (**Figure 4C**). The leaf total phenol content was significantly decreased under CWN compared with CK treatment (**Figure 4D**). The leaf lignin and cellulose contents of *L. formosana* were higher than those of *Q. variabilis*, while the total phenol content of leaves was lower than that of *Q. variabilis*. The interactive effects of nitrogen and water additions significantly affected specific leaf area, total phenols, lignin, and cellulose contents (**Table 2**).

**Table 2:** Effects of species, nitrogen, water, and their interactions on non-structural carbohydrates, water use efficiency, and other physiological properties using three-way ANOVA.

Variable	F_S_	F_N_	F_W_	F_S × N_	F_S × W_	F_N × W_	F_S × N × W_
δ^13^C	**0.010**	**<0.001**	**0.004**	0.123	0.767	0.790	**0.023**
WUE	**0.010**	**<0.001**	**0.004**	0.124	0.770	0.793	**0.023**
Chl a	**0.014**	**<0.001**	0.936	0.984	0.456	**<0.001**	0.146
Chl b	0.603	**<0.001**	0.167	0.591	0.591	**0.003**	0.073
TChl	0.053	**<0.001**	0.682	0.830	0.460	**0.001**	0.103
Chl a/b	**0.003**	0.685	**0.027**	0.188	0.578	0.831	0.155
Leaf NSCs	0.674	0.914	0.707	0.105	0.568	0.537	0.719
LTS	0.829	0.259	0.358	0.096	0.113	0.990	0.405
Leaf starch	0.239	0.074	0.605	0.281	0.280	0.205	0.671
Root NSCs	0.893	0.091	0.743	**0.01**	0.844	0.167	0.583
RTS	0.774	0.061	0.501	0.224	0.300	0.300	0.590
Root starch	0.637	0.391	0.901	**0.005**	0.210	0.241	0.732
Lignin	**<0.001**	0.293	0.776	**0.001**	0.186	**0.026**	0.319
Cellulose	**<0.001**	**0.003**	0.754	0.931	0.541	**0.003**	0.275
Total phenol	**<0.001**	**<0.001**	0.178	0.139	0.382	**0.001**	0.225
SLA	**<0.001**	0.629	0.089	0.628	0.437	**0.005**	0.341

Significant (P < 0.05) effects are presented in bold.

δ^13^C, carbon stable isotope; WUE, water use efficiency; Chl a, chlorophyll a; Chl b, chlorophyll b; TChl, chlorophyll a + b; Chl a/b, chlorophyll a/b; NSCs, non-structural carbohydrates; LTS, leaf total soluble sugar content; RTS, root total soluble sugar content; SLA, specific leaf area.

### Principal component analysis

**Figure 5:**
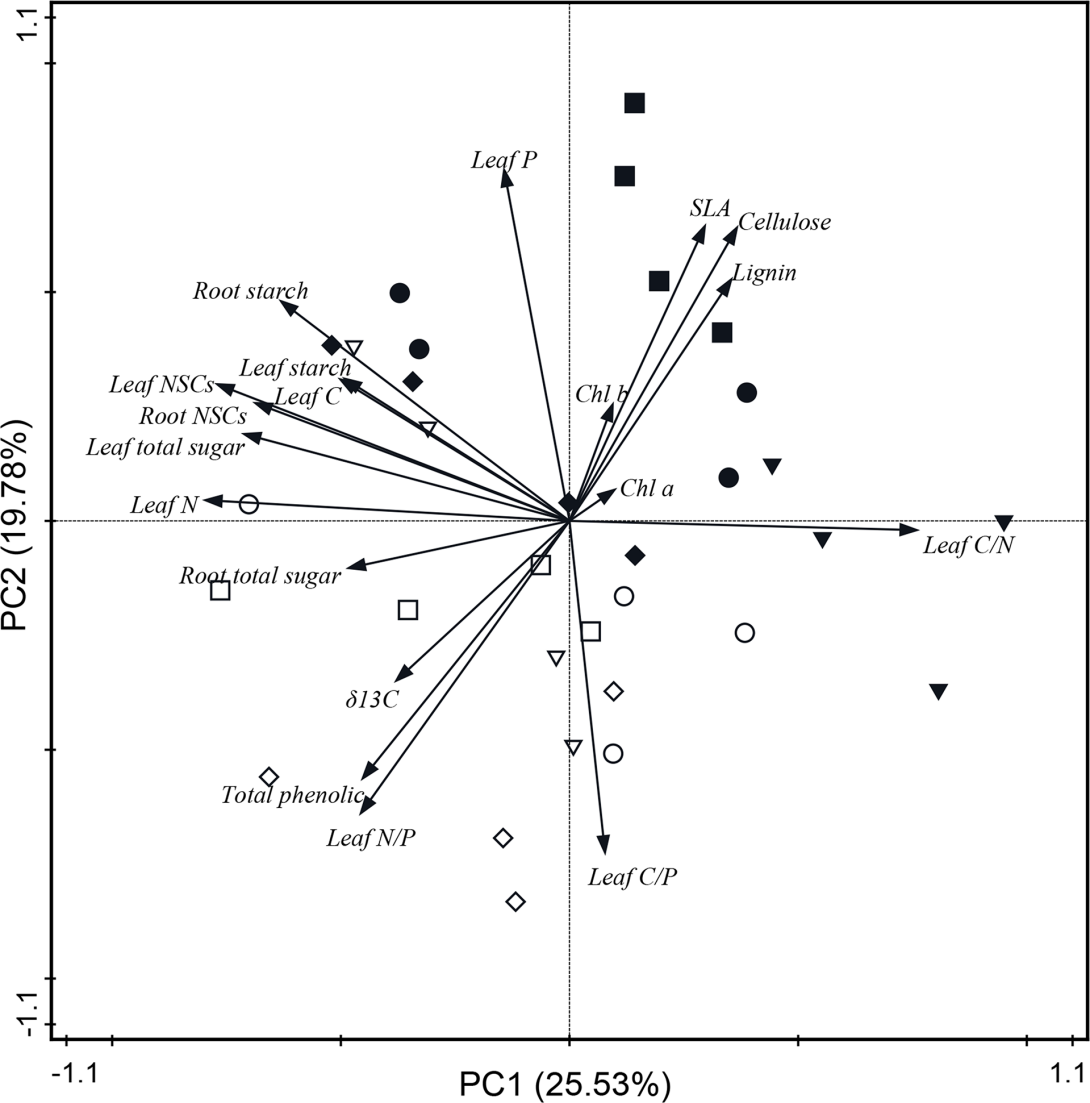
Principal component analysis based on the physiological traits of both *Q. variabilis* and *L. formosana* under canopy N and water additions. CK, control group; CN, canopy N addition; CW, canopy water addition; CWN, canopy N and water interaction; white circle, CK with *Q. variabilis*; white inverse triangle, CN with *Q. variabilis*; white diamond, CW with *Q. variabilis*; white square, CWN with *Q. variabilis*; black circle, CK with *L. formosana*; black inverse triangle, CN with *L. formosana*; black diamond, CW with *L. formosana*; black square, CWN with *L. formosana*; Chl a, chlorophyll a; Chl b, chlorophyll b; SLA, specific leaf area; NSCs, non-structural carbohydrates.

The PCA showed the relationship between leaf physiological traits and the adaptability of trees to changes in N and water addition in *Q. variabilis* and *L. formosana*. Each treatment of both *Q. variabilis* and *L. formosana* was scattered (**Figure 5**), and the PCA for the two tree species alone is shown in the appendix (**Supplementary Figures S2**, **S3**). The PCA model with two components explained 45.31% of the observed total variance. PC1 was strongly influenced by leaf total soluble sugar, starch, NSCs, leaf C and N content, chlorophyll a, C/N ratio, root NSCs, starch, and total soluble sugar. PC2 was strongly influenced by leaf P content, C/P and N/P ratios, δ^13^C, lignin, total phenol, SLA, cellulose, and chlorophyll b (**Figure 5**). In addition, leaf N content showed a positive correlation with NSCs in leaves and roots and leaf WUE, whereas negative correlations were observed with the C/N ratio, SLA, lignin, and cellulose content.

## Discussion

### Long-term water use efficiency response to N and water additions

WUE is an indicator closely related to plant growth under water deficit conditions (Monclus et al., 2006), and water stress increases the WUE of plants (Rahimi et al., 2013; Liu et al., 2015; Yang et al., 2016; Wang et al., 2018). In general, enhanced precipitation increases ambient humidity (Fu et al., 2018) and reduces the WUE (Niu et al., 2011). In this study, increasing precipitation elevated the WUE of *Q. variabilis*, while it did not have a significant effect on *L. formosana*, indicating that different tree species have different water use strategies under precipitation change. An increase in WUE may be related to a drop in SLA or total chlorophyll under increased precipitation. The SLA reflects the ability of the leaf to intercept light, and WUE is negatively correlated with SLA (Wright et al., 1994). In addition, the response of WUE to precipitation is primarily controlled by carbon rather than hydraulic processes (Niu et al., 2011). N is an important component involved in photosynthesis and chlorophyll synthase (Savitch et al., 2002), and the increased content of leaf N implies the enhancement of photosynthesis (Bauer et al., 2004). In this study, canopy N deposition decreased the WUE of the two dominant tree species, and N deposition and increased precipitation had no significant effect on WUE, which was partly consistent with the first and third hypotheses. This suggested that increasing precipitation and N deposition can affect the response of WUE. In the results, N deposition or increasing precipitation had no obvious effect on leaf N content, and there was also no direct association between leaf N and WUE, which suggested that WUE was driven more by abiotic environmental changes than by plant nutrients (Wang et al., 2016).

In addition, the interception of N deposition by the canopy leaves and stems can cause more canopy N accumulation and reduce the N input in soil. N trapped by the canopy is retained or assimilated through the leaf and bark surface (Gaige et al., 2007). Studies have shown that the retention N rate of the plant canopy is 44% at the same N concentration (Liu et al., 2020), and an excessively high N supply reduces a plant’s photosynthetic capacity, which may reduce the plant WUE (Bauer et al., 2004; Rahimi et al., 2013). Previous research has verified that canopy N addition does not affect the carbon assimilation rate of *L. formosana* and *Q. variabilis* (Hu et al., 2021). Moreover, different N forms are absorbed differently by tree leaves with various N uptake strategies—for example, deciduous trees absorbed more ammonium N content than nitrate N in the winter (Ma et al., 2021). In this study, we found that N deposition significantly reduced the soil pH, which affected the soil chemical properties and soil microbes, and reduced the leaf WUE. However, soil pH value had no obvious change under N and water interaction (**Supplementary Table S1**). Shi et al. (2018) indicated that increased precipitation exacerbated the effect of N deposition. The inconsistency with previous studies could be due to differences in latitude region, studied species, and local climate feature.

### Non-structural carbohydrate response to N and water additions

In this study, leaf NSCs, starch, and total soluble sugar concentrations showed less variation with treatments (**Figure 2**), which was consistent with the findings of other studies (Li et al., 2018; Mo et al., 2020; Tang et al., 2020). Previous studies have implied that the N and P levels in leaves can influence the production of NSCs (Xie et al., 2018). Interestingly, only the N addition treatment had an obviously negative effect on the starch, total soluble sugar, and NSC content of the roots of *L. formosana*, which suggested that the response of tree root NSCs to N application was related to tree species (Li et al., 2018). Moreover, the leaf starch of *Q. variabilis* had a negative relationship with the leaf WUE. On the contrary, the leaf total sugar of *L. formosana* was positively related with leaf WUE (**Table 3**). Previous research has shown that fine-root non-structural carbohydrates decrease with increasing N application (Zhu et al., 2021). When plants were under stress, the starch content decreased and total soluble sugar content increased to maintain osmotic changes (Dietze et al., 2014; Hartmann and Trumbore, 2016). In the present study, both root starch and total soluble sugar content decreased. This indicates that plants consume NSC content to promote plant growth based on increased leaf C/N and root respiration, as NSCs have a connection to the respiration of fine roots and can supply energy for the emergence of new roots and nutrient uptake (Zhu et al., 2021). Huttunen et al. (2013) showed that N fertilization reduced the leaf and root NSC concentrations. A meta-analysis showed that N fertilization significantly reduced the total root NSCs but did not affect the leaf NSC content (Mo et al., 2020), revealing that plants distribute large amounts of carbon to organs with the most limited resources (Poorter et al., 2012). Plants allocate more carbon to grow aboveground than belowground, which is driven by carbon allocation patterns when the nutrients are sufficient (Liu and Greaver, 2010; Li et al., 2018). In addition, the decrease in root NSCs may be due to the competition for nutrients between the roots and the microorganisms, as nitrogen fertilization increases soil microbial carbon and soil organic matter (Shi et al., 2018). CWN did not obviously affect the leaf and root NSCs in our study. This suggests that, by altering the retention and absorption of N and water, canopy processes have an impact on the effects of simultaneous N deposition and enhanced precipitation on NSCs.

**Table 3:** Correlation coefficients of long-term WUE and NSC values for *Q. variabilis* and *L. formosana*.

Species		Leaf NSCs	Leaf starch	Leaf TS	Root NSCs	Root starch	Root TS	WUE
*Q. variabilis*	Leaf NSCs	1.000						
	Leaf starch	0.559^*^	1.000					
	Leaf TS	0.885^**^	0.206	1.000				
	Root NSCs	0.581^*^	0.516^*^	0.502^*^	1.000			
	Root starch	0.703^**^	0.767^**^	0.458	0.788^**^	1.000		
	Root TS	0.212	0.068	0.343	0.732^**^	0.210	1.000	
	WUE	-0.324	-0.603^*^	-0.027	0.072	-0.282	0.341	1.000
*L. formosana*	Leaf NSCs	1.000						
	Leaf starch	0.794^**^	1.000					
	Leaf TS	0.812^**^	0.384	1.000				
	Root NSCs	-0.047	-0.428	0.255	1.000			
	Root starch	0.424	0.097	0.508^*^	-0.038	1.000		
	Root TS	0.182	0.012	0.485	-0.165	0.430	1.000	
	WUE	0.450	0.199	0.638^**^	0.120	0.410	0.257	1.000

The single asterisk indicates a significant correlation at P< 0.05 level (bilateral), while the double asterisks indicate an extremely significant correlation at P< 0.01 level (bilateral).

Q. variabilis, Quercus variabilis Blume; L. formosana, Liquidambar formosana Hance; TS, total soluble sugar; NSCs, non-structural carbohydrates; WUE, water use efficiency.

Furthermore, the effect of N addition on tree species NSCs in trees might be related to changes in the content of defense structures (Koricheva et al., 1998; De Long et al., 2015). In this study, N deposition reduced the leaf lignin content of *Q. variabilis*, which would be linked to the higher allocation of C to plant growth rather than structural carbohydrates. N addition reduces the total phenol content in plants, thus affecting plant growth (Lu et al., 2008). CWN did not affect the leaf and root NSCs in our study (**Figure 4**). This suggests that, by altering the retention and absorption of N and water, canopy processes have an impact on the effects of simultaneous N deposition and enhanced precipitation on NSCs. In this study, CWN decreased the total phenol content of the two tree species (**Figure 4D**). The leaf N/P ratio of *Q. variabilis* was above 16 under N application and increased precipitation, which indicates P-limited biomass. Then, N and water interaction affected tree defense by changing the leaf nutrient limit, as reduced plant nutrient limitation leads to a lower C allocation to secondary metabolite production (Bryant et al., 1983). Factors such as tree taxonomic type, leaf habit, and tree ages can affect plant responses to the driver of external factors (Ramírez-Briones et al., 2017; Mo et al., 2020; Tixier et al., 2020). Moreover, the root NSCs of the two dominant tree species had no significant change under CWN treatment, indicating that increasing precipitation can alleviate the effect of N addition on root NSCs. This finding supported our hypotheses, which stated that N addition decreases the root NSC concentration of tree species, while increased precipitation mitigates the effect of N deposition. The physiological characteristics of the two dominant tree species had different responses to the nitrogen–water interaction, which could be due to their growth strategies to external disturbances.

## Conclusion

The leaf WUE and NSCs of two dominant tree species showed different responses to canopy N deposition and water addition in a warm temperate forest. Elevated N deposition reduced the root NSC (total soluble sugar and starch) concentration of *L. formosana*, while it did not obviously change the leaf NSCs of the two dominant tree species. N deposition decreased the leaf WUE of *Q. variabilis* and *L. formosana*. Increased precipitation showed a positive effect on the leaf WUE of *Q. variabilis*. However, the N–water interaction did not obviously affect the leaf WUE and NSC contents of the two dominant tree species. This presented the dominant tree species regulated growth traits when faced with changes in external factors, such as enhanced tree adaptability by altering the tree NSCs and long-term WUE. Additionally, the leaf WUE was affected by changes of the abiotic environment rather than leaf nutrients. When examining the impacts of N deposition on the physiological responses of the dominant tree species, it is crucial to consider the canopy nitrogen and water interactive effects, as the two natural processes frequently occur at the same time. The simulated canopy N deposition and precipitation addition provided a new method for researching the physiological responses of dominant tree species to global change.

## Data availability statement

The original contributions presented in the study are included in the article/**Supplementary Material**. Further inquiries can be directed to the corresponding authors.

## Author contributions

XJ and MS had the main responsibility for data collection, analysis, and writing. YQ, ML, and LM contributed to data and manuscript preparation. MS and SF (the corresponding authors) had the overall responsibility for experimental design and project management. All authors contributed to the article and approved the submitted version.

## Acknowledgments

This work was supported by the National Natural Science Foundation of China (no. 31800369 and no. U1904204), the State Scholarship Fund of China, and the Innovation Scientists and Technicians Troop Construction Projects of Henan Province (no. 182101510005). We are also grateful to the Henan Dabieshan National Field Observation and Research Station of Forest Ecosystem for supporting the fieldwork.

## Conflict of interest

The authors declare that the research was conducted in the absence of any commercial or financial relationships that could be construed as a potential conflict of interest.

## Publisher’s note

All claims expressed in this article are solely those of the authors and do not necessarily represent those of their affiliated organizations, or those of the publisher, the editors and the reviewers. Any product that may be evaluated in this article, or claim that may be made by its manufacturer, is not guaranteed or endorsed by the publisher.
