# Supplementary material for: Long-term water use efficiency and non-structural carbohydrates of dominant tree species in response to nitrogen and water additions in a warm temperate forest
Source: Front Plant Sci. 2022 Nov 7;13:1025162. doi: 10.3389/fpls.2022.1025162 (PMC9676439; doi:10.3389/fpls.2022.1025162)

Table S1. Soil basic biochemical properties under nitrogen and water addition conditions.

	CK	CN	CW	CWN
SOM ($g \cdot kg^{-1}$)	46.65±11.51 b	98.66±20.53 a	46.17±10.60 b	64.07±4.87 ab
TN ($g \cdot kg^{-1}$)	1.06±0.19 a	1.27±0.05 a	0.99±0.15 a	1.31±0.06 a
TP ($g \cdot kg^{-1}$)	0.36±0.06 a	0.40±0.04 a	0.33±0.05 a	0.44±0.04 a
AN ($mg \cdot kg^{-1}$)	124.71±23.13 b	193.77±18.81 a	125.27±23.06 b	177.53±15.31 ab
AP ($mg \cdot kg^{-1}$)	25.14±3.50 a	25.84±0.69 a	22.78±3.94 a	29.64±0.66 a
pH value	4.40±0.12 ab	4.14±0.07 b	4.60±0.08 a	4.19±0.06 b
Urease ($mg\ NH_3\text{-}N\ g^{-1}\ soil\ d^{-1}$)	26.79±3.46 a	36.52±2.97 a	26.69±3.57 a	33.02±1.60 a
Sucrase ($mg\ Glucose\ g^{-1}\ soil\ d^{-1}$)	214.71±28.51 a	263.50±9.38 a	210.26±26.30 a	250.26±9.31 a
Nitrate reductase ($mg\ NO_2\ g^{-1} \cdot d^{-1}$)	8.46±1.39 a	10.52±1.06 a	7.98±1.62 a	11.07±0.98 a
MBC ($mg \cdot kg^{-1}$)	357.49±25.27 a	416.89±16.19 a	357.45±22.28 a	390.85±12.07 a
MBN ($mg \cdot kg^{-1}$)	59.10±3.45 a	63.06±2.38 a	58.19±4.43 a	64.60±2.09 a

In the same row, different lowercase letters mean significant differences under four treatments according to ANOVA with LSD tests ($P < 0.05$). CK, control treatment; CN, canopy N addition; CW, canopy water increase; CWN, canopy water and N addition. SOM, soil organic matter; TN, soil total nitrogen; TP, soil total phosphorus; AN, available nitrogen; AP, available phosphorus; MBC, soil microbial biomass carbon; MBN, soil microbial biomass nitrogen.

Figure S1. Canopy nitrogen addition and water increase device, a triangular iron tower with a height of 35 m, was built in the center of each circular quadrat.

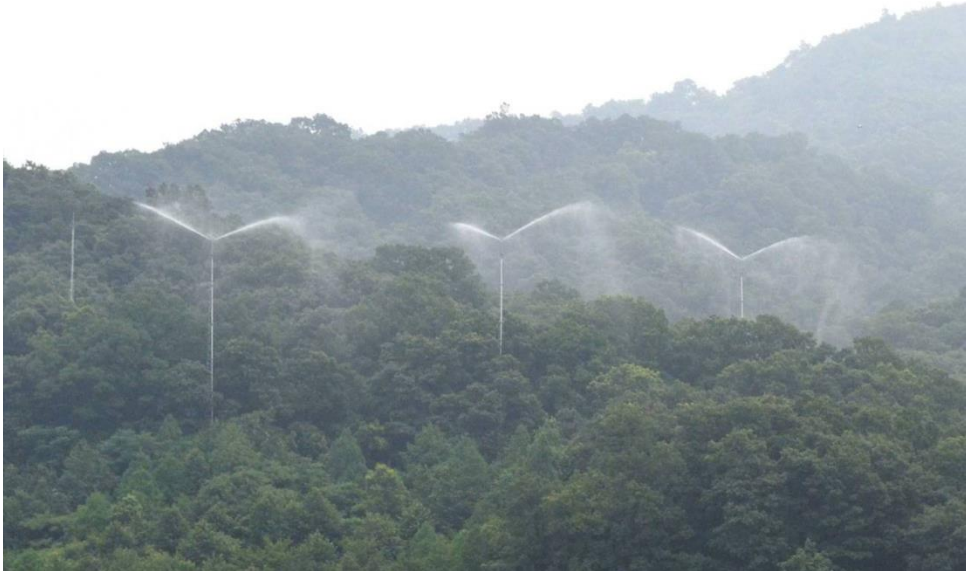

Figure S2. The PCA based on physiological traits of *Q. variabilis* under N and water addition conditions. CK, white circle; CN, white inverse triangle; CW, white diamond; CWN, white square. Chl a, chlorophyll a; Chl b, chlorophyll b; SLA, specific leaf area; NSCs, non-structural carbohydrates.

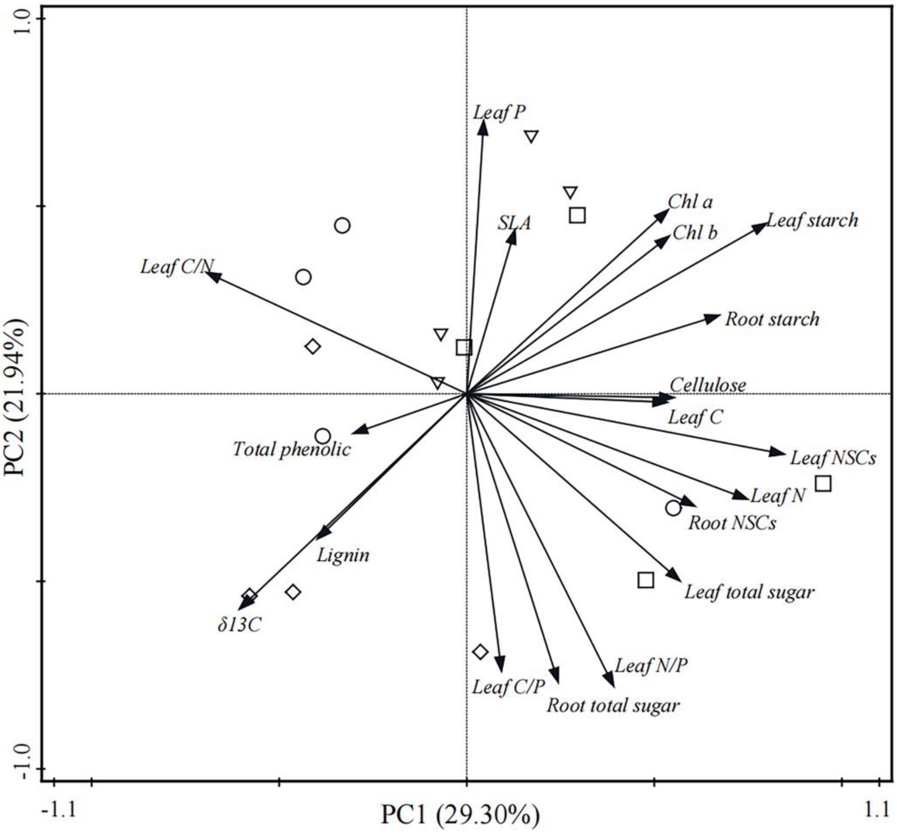

Figure S3. The PCA based on physiological traits of *L. formosana* under N and water addition conditions. CK, black circle; CN, black inverse triangle; CW, black diamond; CWN, black square. Chl a, chlorophyll a; Chl b, chlorophyll b; SLA, specific leaf area; NSCs, non-structural carbohydrates.

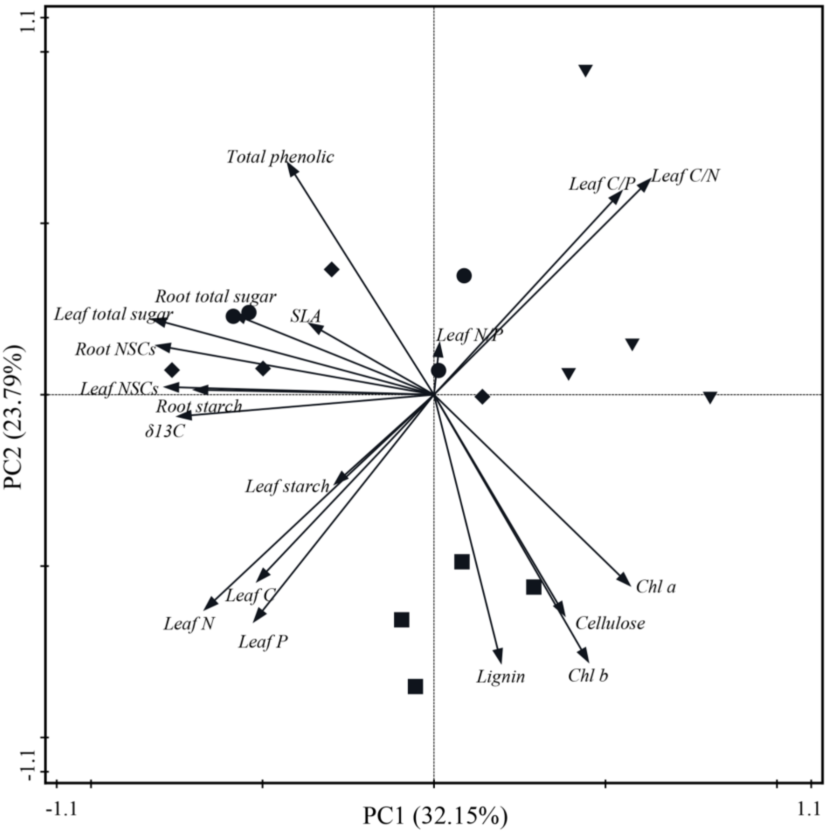

Supplement: Supplementary file 1 [file DataSheet_1.pdf]
